# Sustaining a “culture of silence” in the neonatal intensive care unit during nonemergency situations: A grounded theory on ensuring adherence to behavioral modification to reduce noise levels

**DOI:** 10.3402/qhw.v9.22523

**Published:** 2014-03-18

**Authors:** S. Swathi, A. Ramesh, M. Nagapoornima, Lavina M. Fernandes, C. Jisina, P. N. Suman Rao, A. Swarnarekha

**Affiliations:** 1 Department of Otolaryngology Head & Neck Surgery, Bangalore, India; 2 CMR Institute of Management Studies, Bangalore, India; 3 Department of Neonatology, St John's Medical College Hospital, Bangalore, India

**Keywords:** Grounded theory, noise, NICU, operant conditioning

## Abstract

The aim of this study was to generate a substantive theory explaining how the staff in a resource-limited neonatal intensive care unit (NICU) of a developing nation manage to ensure adherence to behavioral modification components of a noise reduction protocol (NsRP) during nonemergency situations. The study was conducted after implementation of an NsRP in a level III NICU of south India. The normal routine of the NICU is highly dynamic because of various categories of staff conducting clinical rounds followed by care-giving activities. This is unpredictably interspersed with very noisy emergency management of neonates who suddenly fall sick. In-depth interviews were conducted with 36 staff members of the NICU (20 staff nurses, six nursing aides, and 10 physicians). Group discussions were conducted with 20 staff nurses and six nursing aides. Data analysis was done in line with the reformulated grounded theory approach, which was based on inductive examination of textual information. The results of the analysis showed that the main concern was to ensure adherence to behavioral modification components of the NsRP. This was addressed by using strategies to “sustain a culture of silence in NICU during nonemergency situations” (core category). The main strategies employed were building awareness momentum, causing awareness percolation, developing a sense of ownership, expansion of caring practices, evolution of adherence, and displaying performance indicators. The “culture of silence” reconditions the existing staff and conditions new staff members joining the NICU. During emergency situations, a “noisy culture” prevailed because of pragmatic neglect of behavioral modification when life support overrode all other concerns. In addition to this, the process of operant conditioning should be formally conducted once every 18 months. The results of this study may be adapted to create similar strategies and establish context specific NsRPs in NICUs with resource constraints.

Fifty dB (A) is the recommended ambient noise level in the neonatal intensive care unit (NICU) (Philbin, Robertson, & Hall, 1999; White et al., 2013). Maintaining noise levels within 50 dB (A) is associated with reduced risk of developing noise-induced adverse effects (Philbin et al., 1999). Noise above the recommended level has short- and long-term effects. The short-term effects are tachycardia, tachypnea, and hypoxia. The long-term effects are higher risk of developing mild hearing loss, retardation of intelligence development, periventricular hemorrhage, and leukomalacia (Bremmer, Byers, & Kiehl, 2003; Li, Jiang, Gan, Zhou, & Chen, 2009; Li & Steyger, 2009; Wachman & Lahav, 2010). In view of this, noise reduction protocols (NsRP) comprising behavioral and infrastructural modifications are recommended as the standard of care in the NICU (Chaudhari, 2011; Ramesh et al., 2009). Active and passive noise control methods are the two main methods to reduce noise. Passive control is based on creating barriers as well as reducing reverberations. It is mainly for high frequency noise which has a shorter wavelength. Active methods create anti-noise, which is sound in the opposite phase and cancels the low frequency noise (Altuncu et al., 2009; Bellieni et al., 2003; Bistrup, Babisch, Stansfeld, & Sulkowski, 2006; Kim & Lee, 2002). In a setting such as NICU, activity-generated noise is an important component which needs to be controlled. Creating partitions between each station is an effective method to contain the noise. This strategy is not feasible in resource-limited settings because it increases the cost of care. Behavioral modification of staff activity is the most cost-effective strategy in these settings (Ramesh et al., 2009). If these measures are implemented in the design stage of creating the NICU, it may be followed as a routine. If they are introduced at a later stage, they are not effective beyond 18 months, even if the staff members are conditioned (Ramesh et al., 2012).

In 2007, a NsRP was established in our NICU. It consisted of behavioral and structural modifications. Infrastructural modifications comprised two main components: reducing the transmission of noise from outside by barriers, and limiting the reverberations of internal noise. These measures were permanent, as it was incorporated into the structural design of the NICU. The key behavioral modifications to reduce noise were speaking in low tones, avoid shouting across the room, holding discussions in a separate room, handling of trays and metallic objects gently, keeping the phone volume low, and tuning the alarms to emit a maximum of 55 dB. Following this, we conditioned the staff by displaying the noise levels on a board and gave regular positive and negative feedback for 6 months. This process is called operant conditioning. After 18 months, we observed that the effects of the conditioning had waned and there was a need to recondition. The main reason for waning of the conditioning was the highly dynamic NICU environment in terms of activities of the staff. The routine starts with rounds of nursing staff who take over from the night shift from 7:30 AM to 8:30 AM. This is followed by doctor's rounds from 9:30 AM to 12:30 PM. At 1:30 PM, nursing staff in the second shift take over after brief rounds. At 4 PM, the doctors have a short review of the progress since morning rounds. At 7:30 PM, the nurses in the night shift take over with a brief round. Every day, for 2 h, fathers are allowed to visit the neonate. This routine is unpredictably interrupted by a series of emergency situations which arise due to neonates suddenly getting sick. During emergencies, it is not possible to follow any of the NsRP measures. The NsRP was intended to reduce noise during nonemergency situations.

On reviewing literature, we found studies had measured efficiency of NsRPs (Daniele, Pinheiro, Kakehashi, & Balieiro, 2012; Stretcher & Rosenstock, 1997). But there was a lack of studies that described the concerns of the NICU staff members while maintaining reduced noise levels in the dynamic environment of the NICU and how they deal with them. The intention of this study is to generate a substantive theory to explain how NICU staff deal with these concerns. The results could be used by other NICU managers in resource-limited settings to adopt similar strategies and optimize the NsRP.

## Method

This study intends to generate a substantive theory, to explain how the staff in a resource-limited NICU address their concerns while maintaining reduced noise levels. Hence, a theoretical approach of symbolic interactionism and pragmatism was adopted. We followed the principles of reformulated grounded theory to create the substantive theory (Strauss & Corbin, 1990, 1998) but maintained faithfulness to the fundamental tenets of systematics in methodology and constant comparative method as described by Glaser and Strauss (Glaser & Strauss, 1967). Symbolic interactionism may be summed as social interactions shaping the meaning of phenomena and events (Blumer, 1969). Pragmatism is to unify intelligent thoughts, logical method, and practical actions, and to apply it to experiences (Plummer, 2001).We exercised “disciplined restraint” to be unprejudiced while collecting and interpreting data. Unlike the classical approach, the reformulated approach gives an action pathway for NICU managers, where concerns regarding maintaining the NsRP can be addressed.

### Participants

The study was conducted in the level III NICU of a tertiary care hospital in south India. The NICU can accommodate 36 neonates. As the researchers (except the first author: SS) were involved in establishing and implementing the NsRP, we adopted theoretical sampling instead of free sampling. Initially, the nursing staff (20 trained nurses) and neonatology consultants (four consultants) were included; based on the data from the initial few interviews, we included the nursing aides (six aides) and neonatology residents (six residents) also. The age of the staff nurses ranged between 25 and 45 years whereas that of the consultants ranged from 29 to 55 years. The work experience of the staff nurses in the NICU ranged from 10 days to 9 years with a median experience of 6 years. There were four males and 32 females. By including members from all categories in the NICU, optimization of variation in data was achieved which is a prerequisite for data in grounded theory.

### Procedure

Face-to-face interviews were conducted with all staff members of the NICU. Four group discussions were conducted with all of the 20 staff nurses and 6 aides. Interview and moderator's guides were developed by the Delphi method. A set of questions and probes was framed to explore how the staff in the NICU addressed their concerns while maintaining reduced noise levels by behavioral modifications. This was circulated among a set of experts comprising a neonatologist, an otolaryngologist, an audiologist, and a social scientist who were involved in setting up the NsRPs. Their comments were used to modify the questions. The guide was pilot tested during the first five interviews and redundant items were deleted. The interviews were conducted in the NICU at a time convenient to the participants. Care was taken to ensure privacy and confidentiality. Repeat interviews were conducted if the first was interrupted. This occurred on three occasions due to some other activity. The average duration of the interview was 30 min. The interviews were not audio taped. After the interview, the researcher noted the salient aspects and quotes. The transcripts were not returned to the participants for comment or corrections but clarifications were sought verbally if any aspect of the transcript was not clear. The group discussions were conducted in a classroom outside the NICU. The moderator was a medical resident. A trained social scientist, with experience in conducting focus group discussions, assisted her and plotted the sociogram. The sociogram gave a visual representation of the dynamics of the group discussion. The entire discussion was audio taped with informed consent of the participating members. Each discussion lasted approximately for 45 minutes. The audio files were subsequently transcribed and coded. Clarifications about some aspects of the transcript were made after discussion with the participants. Any questions or wrong notions of the group were addressed at the end.

The participants were familiar with the researchers prior to the study, because the researchers (except the first author, SS) had worked with the NICU staff members during implementation of NsRP. The staff members were aware that the purpose of the study was to improve standard of care in the NICU. The researcher who interviewed the participants and facilitated the group discussion was a medical resident and did not have any managerial role or position of authority. To that extent, we may consider her as “independent and unbiased.”

### Analysis of data

Data analysis was done in accordance with the reformulated grounded theory approach, which was based on inductive examination of textual information. We simultaneously collected, coded, and analysed the data. The decision to collect the next set of data was guided by the developing theory (Glaser, 1992; Glaser & Strauss, 1967). The group discussion and interview transcripts were read thoroughly by SS and RA. They were coded inductively by the two authors independently. Hierarchical coding was done: initial open coding, conceptual coding, and theoretical coding. In the first stage of analysis, “What are the concerns while maintaining reduced noise levels” was in the background. The next level of analysis looked at “How does the staff deal with these concerns.” The coders compared the coding schemes and resolved any differences in the coding. Detailed memo writing was done for each code and category to look for comparisons and relationships. N vivo version 9 was used to classify the nodes as free and tree nodes. Coding densities were used to identify recurrent themes. Finally, we conceptualized the concerns and strategies and gave them appropriate names. The main concern that emerged was ensuring adherence of staff to behavioral modification components of NsRP. This was managed by creating strategies to “sustain a culture of silence in NICU during nonemergency situations” (core category). Other categories were linked to this by pattern identification to generate a theory. There was theoretical sensitivity, as the researchers (except SS) were involved in the establishment of the NsRP.

Quality of data in terms of trustworthiness, concordance between data and result, and transferability was ensured (Bertero, 2012; Fagerberg, 2012; Hallberg, 2013). The techniques of respondent validation of the results were used as a method of triangulation to ensure trustworthiness and match between data and interpretations. All the categories of staff were interviewed. Four group discussions were done to get any new views. This ensured data saturation. The quality of the grounded theory was examined in terms of fit, relevance, work, and modifiability.

### Ethical considerations

The study methods were reviewed and approved by the Institutional Ethical Review Board (Ref No. 008/2011). Written informed consent was taken for participation in the study. No honorarium was paid. The participants were informed that participation was voluntary and they had a right to withdraw at any time without any prejudice. The transcripts were kept confidential in a secure place with restricted access.

## Results

**Figure 1:**
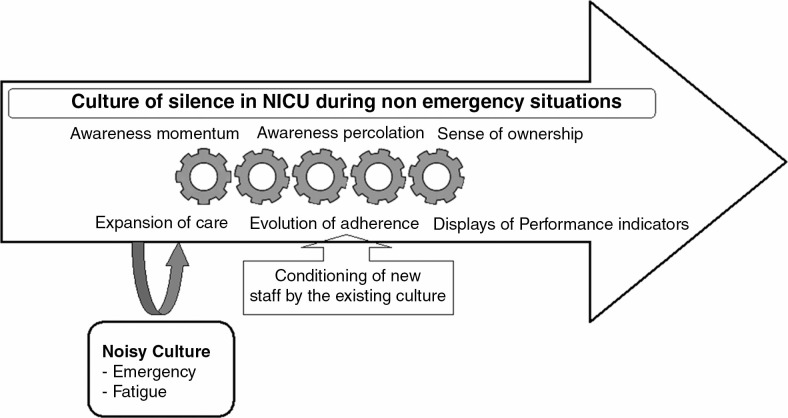
A substantive theory was generated, explaining how the main concern of the NICU staff to ensure adherence of staff to behavioral components of noise reduction protocol during nonemergency situations was resolved by sustaining a “culture of silence” in neonatal intensive care unit during nonemergency situations (core category). The strategies, related to the core category, were building awareness momentum, causing awareness percolation, developing a sense of ownership, expansion of caring practices, evolution of adherence, and displaying performance indicators.

Based on the interviews and group discussions with the NICU staff, we generated a theory to describe the strategies they used to deal with their concerns while ensuring adherence to behavioral modification components of the NsRP. The implementation of the NsRP is a continuous process. First behavioral and infrastructural modifications are established. These are later modified by the prevailing conditions in the NICU based on feasibility issues and views of the NICU staff members about the NsRP. The staff dealt with their concerns by creating strategies that aid in “sustaining a culture of silence in NICU during nonemergency situations” (core category). The strategies that emerged were building awareness momentum, causing awareness percolation, developing a sense of ownership, expansion of caring practices, evolution of adherence, and displaying performance indicators. Figure 1 shows schematically how these strategies sustained a “culture of silence” which ensured that existing staff adhered to the NsRP and new staff got conditioned by the existing culture.

### Sustaining a “culture of silence” in the NICU during nonemergency situations—the core category

To achieve reduced noise levels, the staff has implemented an NsRP. The important components are speaking in a low tone, avoiding shouting across the room, holding discussions in a separate room, handling of trays and metallic objects gently, avoiding using personal mobile phones, keeping the phone volume low, and tuning the alarms to emit a maximum of 55 dB. This patchwork of behavioral practices creates what can be conceptualized as “culture of silence.” The “culture of silence” creates an ambience which not only influences the existing staff but also the new people who walk into this environment. Doctors and personnel from other departments are influenced by the reduced noise levels. This makes them tone down their voices. During emergencies, there are temporary spikes in the noise levels. This is due to “pragmatic neglect” of behavioral modification when life support concerns override all other concerns. After the emergency situation passes, the ambience of reduced noise returns again. This can be seen as the self-conditioning and self-restorative property of the noise-reduced environment created as a result of the “culture of silence” which is seen in the following quote.

I used to work in the medical and surgical intensive care unit before. The general noise levels were quite high. So we did not bother much. On coming here I could sense an ambience of silence. This has made me to speak softly. When the noise is less we speak softly and when noise is high we speak loudly. (N3)

During the establishment phase of the NsRP, some of the staff acted as monitors to ensure adherence to the behavioral components. This was followed by a 6-month period of operant conditioning. Following this, various strategies were used by the staff to sustain the “culture of silence.” It is the sustained presence of this culture which has ensured adherence to NsRP. The following sections describe the strategies.

### Building an awareness momentum

The group discussions revealed that during the establishment phase of NsRP, a set of informative sessions were conducted to create awareness about the harmful effects of noise. These sessions have created a body of knowledge in the minds of the staff. Specific effects such as “increasing heart rate and breathing pace” have affected their understanding of harmful effects of noise. The awareness has ensured that the staff members recognize the effects and get a real sense of the harm caused by this invisible pollutant. One of the senior staff members said:

In sick babies we are aware that high noise levels can increase heart and breathing rate which is not good for the baby. Noise is harmful also for premature babies … I learnt in the classes taken by one of the doctors …. It was surprising to know that high noise can be so serious an issue. I used to think that it is just a cause for irritation. (N1)

The quantum of awareness about behavioral modification exists in a general as well as a specific sense. They have also imbibed the fact that high noise specifically deters the recovery of the neonates by hindering the physical as well as mental well-being. The body of knowledge created during the establishment phase of the NsRP can be seen as a push or momentum given to the process of “sustaining a culture of silence.” The following quote illustrates the specific and general aspects of quantum of knowledge.

Different types of patients, babies having different conditions, so it will disturb the mental and physical aspects of the baby. We don't use our individual mobile phones while in the NICU. We have to be gentle, not to make much noise while handling the machines, door locking. (N8)

However, over time, the momentum slows, requiring another cycle of formal sessions and reconditioning. We found out that it is a good idea to explore the views of the staff on why the “culture” has faded and potential roadblocks that may contribute to a “noisy culture.” This is clear from the following quotes:

And we need discussions like this every now and then. This will help in reinforcing the noise reduction habits. (N4)

I agree that silence was maintained for at least 18 months. But there is a need to start the cycle of conditioning formally every 18 months so that the conditioning and motivation is refreshed. (D2)

There should be an on-going effort to re-enforce and remind staff about the issue periodically and constant reminders are necessary. (D3)

### Causing awareness percolation

As new personnel join the NICU, they get inducted into the environment where there is a “culture of silence.” Over time, due to social interactions, the awareness regarding the NsRP among the existing staff gets passed onto the new staff and they too get aligned to the “culture.” In addition to the percolation of knowledge about general aspects of noise being harmful, the specific aspects of NsRP behavioral modification has also percolated to the new staff as seen in the following two quotes.

But after coming to this place I have become aware that high noise is harmful and may even impede the recovery of the babies. (D3)

We should avoid shouting in the NICU. We should talk softly and handle objects carefully. The doctors also discuss about the babies in a separate room. (N4)

The awareness percolation is aided by the existence of certain visual cues and practices. The sound level meter used by the staff to measure noise during each shift reminds the staff that noise needs to be reduced. The audiology staff coming in for routine hearing screening adds on to the fact that noise control is important.

I just came, I'm not very sure of it, but I know that there is a decibel meter used to measure sound in each room. (N6)

Every shift, we record the sound levels in the NICU, that is, in the morning, afternoon and evenings. Also, every Friday the audiologists come for testing the babies. This reminds us that noise has to be reduced. Usually in other places, noise does not occur as real. (N6)

### Expansion of caring practices

The nursing staff members have not felt the behavioral modification measures to be a burden to the already existing workload. Instead, they see it as an add-on to the care they are providing to the babies. The staff experience that noise reduction makes the baby comfortable. The nurses after becoming aware of the effects of noise on the neonates recognize vulnerability of the sick neonates to loud noise. They see reduced noise as “redefined care” being given to them. Their humaneness toward the sick neonates is reflected when they try to pacify the babies after noise spikes in the NICU environment. It was seen that “It makes a difference when noise is controlled as well as the babies are pacified after noisy activity” is a recurring theme in all the quotes.

What I have seen is, if the nurses speak to the baby, the baby is much calmer. If they just leave the baby when the alarm is going off the babies are restless … the moment a nurse is around or someone comes, the baby gets calmed. (N2)

Low noise is required to give patient care properly, otherwise … patient will get disturbed because of the sounds. Most of our patients are sick with different conditions. (N8)

It makes a lot of difference when a nurse is talking to the baby to pacify after the alarms go off. (N1)

The nurses have also come up with additional measures which were not in the NsRP, to provide more comfort to the babies in a noisy environment. This is a clear evidence of the caring attitude as well as the fact that behavioral modifications is seen as expansion of care being provided to the neonates.

There's a normal way of talking to the baby, positive assurance that you give to the baby … means soft speaking. soft noises and things like that … if the sound is to the extreme, it can affect the babies’ development. (N10)

### Evolution of adherence

The staff in the NICU during the establishment of the NsRP was conditioned to follow the behavioral modification measures. The new staff members who join the NICU also get conditioned by the existing staff. This happens passively by the new staff observing the behavior. The existing staff also actively aligns the new staff to keep noise levels low by adhering to the NsRP. During this active process, the members are careful not to be “judgmental” about the whole process. This prevents invalidation of the new staff and brings about change in an informal and friendly manner. Three newly recruited nursing staff had the following to say:

After working in the Medical ICU and joining here, first one week, I really struggled not to raise my voice. People used to nudge me from the back and ask me to keep quiet, I'm still learning. The thing is there is a difference. Where I used to work, people don't care about how much noise they make, here people are softer; the noise is overall less, so you tend to become less noisy. (N1)

I was not here when the NsRP was established but as soon as I came 6 months ago, I was instructed to be careful and not make noise. Everyone seems to be motivated to follow NsRP as well as tell others to follow the same. (D3)

This process of alignment of the new staff actively and passively in a nonjudgmental manner can be conceptualized as “evolution of adherence” of new staff to behavioral modification components of the NsRP.

### Displaying performance indicators

The NICU staff has created three methods to augment the performance of the NsRP. Visual cues, reading “rituals,” and visibility exercises. The process of people measuring noise levels and displaying it transforms noise to a “tangible element.” This exercise plays a role in continually conditioning the staff as articulated in the following quote.

Every day we observe someone measuring the noise levels during all the shifts. After the noise levels are recorded, it is displayed on a board in the nursing lobby. This keeps reminding us that we need to keep noise levels low. Sometimes when the levels are good we feel happy. (N5)

The NICU displays a set of “10 rules for the little angels” which explains methods to reduce noise levels. The staff members are requested to read them at regular intervals. The staff have expressed that these reading rituals reinforce their commitment to reduce noise levels. The following quotes illustrate the effect of these visibility exercises on their performance.

A set of 10 rules to keep noise low is displayed along with the board displaying the noise levels. The staffs are encouraged to read this to remind us of the fact that we need to stick to these rules. (D1)

Another factor that augments the performance is appreciation of the NsRP by faculty from other centers. In addition, the staff members who have been on exposure visits to other NICUs in India have seen similar protocols being followed. The following quotes illustrate the effect of these visibility exercises on their performance.

I've visited 20 NICUs all over India. I think most NICUs are aware of the effect of noise on the neonates; awareness was there and I think it's a good initiative on their part. But comparatively we're doing much better I feel, and that should be an encouragement to all of us here. (N1)

We have had faculty visiting from other universities and also from countries such as United States, Europe, Middle East and others. All of them were happy to see that we have this NsRP. Some from advanced settings have even said that they do not have such a streamlined NsRP. We feel proud that we are having these measures in our NICU. It makes us feel appreciated. In a stressful place like the NICU it is very fulfilling. (N8)

These strategies are effective performance boosters. In a stressful environment like the NICU, these methods can encourage the staff to adhere to behavioral components of the NsRP.

### Developing a sense of ownership

The NICU staff feels that NsRP is something that got created by an internal initiative to improve quality of care. Because of this, they do not want any external authority to monitor them. The belongingness to the NsRP is reflected in their constructive suggestions for further improvement. The sense of ownership by the NICU staff toward the NsRP is a “catalytic factor” which sustains the “culture of silence.” These quotes bear evidence to this fact.

We don't need anybody from outside to police us. We did have some of us who acted as the noise monitors during the initial days when the protocol was put in place. (N8)

“There is a requirement for more posters and we need discussions like this every now and then. This will help in reinforcing the noise reduction habits.” We can use visual alarms instead of the audible alarms. (N3, N4)

### Pragmatic neglect during emergency situations—Noisy emergency culture

In an emergency situation, the concern to save life overrides all other concerns. The staff members choose to neglect the NsRP and ensure that the required assistance is sought by forcefully and loudly communicating to the other staff as seen in the following quote.

When a baby crashes, we need to immediately act. There will be shouting across the room, lot of people rushing in, obviously in such cases, noise is a lesser priority, can't be helped. (N6, N10, N11)

A lot of sick neonates, at any point of time, increases the workload resulting in stress and fatigue. This adds up to the noise during emergencies as inferred from the following quotes.

That's what I've seen, when the girls are very busy, the noise is high, otherwise in the normal usual way, everybody is in low volume mode but when the stress level go up, voice goes up. (N1)

Sometimes the work can get hectic, when there are a lot of sick babies. During those times when the alarms go off erratically it can be stressful and irritating. Based on the stress level noise often goes up. (N11)

Reduced human resources inside the NICU tax the current staff members. The workload and the stressful environment can in turn exaggerate the work pressure, especially in acute emergencies. Added to this, the need to maintain “sterile conditions” while performing routine care hinders immediate response to noise-generating situations.

In the ventilator room, we have one nurse for four babies. Ideally, it should be one nurse for one baby. When they are feeding one baby, if some other baby monitor beeps, because the nurse is in sterile gowns and in the middle of feeding, she is not able to put it off and it keeps on beeping. In the stable room, one nurse has to look after nine babies. If one baby starts crying, the others babies also start. She'll start with baby 1. After she starts feeding them, cleans them up and finishes baby 1 to 9, baby 1 starts crying again …! (N1)

During periods of excess work, because of fatigue, the staff get used to alarms that go off erratically and this again contributes to the “noisy culture” in these phases. As seen in the quotes below, the staff get a feeling of suffocation and irritation at times and ignore the usual noise reduction measures of the NsRP.

A feeling of suffocation comes when asked to whisper always. When the work load is heavy, when there are many sick babies we feel a need to talk to others while doing the work. If we are not able to do that, it can get very difficult at times. (A6)

You see from the doctors to the aides who clean, they're all are aware that we have to maintain this, at times, for example, yesterday, we were with the other baby, the alarm was going on madam pointed to, sister see this alarm is going on. I mean it is not that we are not at all aware, maybe we give concentration to something else, and we completely neglect what is happening. (N4)

Thus a definite “culture of silence” exists only during periods of relative “normalcy” The NsRP becomes relevant only in such situations.

## Discussion

The present study has developed a substantive theory to explain how the staff of a resource-limited NICU deal with their concerns while maintaining reduced noise levels. Review of relevant literature in this area showed a few studies that have explored the issues with implementation of the NsRP. None of them have attempted to construct a theoretical model to explain how the staff deal with concerns while maintaining reduced noise levels. Our model is grounded in the data from interviews and focus group discussions with the NICU staff. We have modeled the theory on the tenets of symbolic interactionism. Symbolic interactionism consists of three premises. The first premise is that, action of human beings toward things is determined by the meaning they give to them. The second premise is that, the meaning of such things is derived from the social interactions between human beings. The third premise is that, these meanings are modified by the experiences while acting on these things (Blumer, 1969). All these aspects are very relevant in the context of the NICU staff members following the behavioral modifications enshrined in the NsRP. Noise is not visible and the human ear gets adapted to high noise levels and stops perceiving it as loud. Nevertheless, noise continues to cause harm. The first premise of symbolic interactionism is relevant here because compliance to NsRP is determined by the meaning attributed to the behavioral modifications during the establishment phase. These meanings are modified by their interactions with fellow colleagues which is the second premise of symbolic interactionism. The staff members in the NICU have their own concerns while keeping noise levels low, especially in nonemergency situations and develop strategies to deal with them. These strategies serve to condition the new staff members who join the NICU. This is the third premise of symbolic interactionism.

Their main concern among the NICU staff was to ensure adherence to behavioral modification components of an NsRP during nonemergency situations. The staff used the strategy of “sustaining a culture of silence in NICU during nonemergency situations” (core category) which ensured adherence to the behavioral modifications. Figure 1 shows the schematic representation of the model. The methods used to sustain a “culture of silence” were building awareness momentum, causing awareness percolation, expansion of caring practices, evolution of adherence, displaying performance indicators, and developing a sense of ownership.

In our study, we discovered that the NICU staff members have been motivated by the concept of maintaining a quiet NICU during nonemergency times to enhance the recovery of neonates. The self-motivation is the result of the group norm that has been created by the initial conditioning phase of the NsRP. Group norms has been identified as an important factor influencing NICU practices (Thomas, Sherwood, Mulhollem, Sexton, & Helmreich, 2004). Another study that has used a qualitative research with a sociohistorical approach has also concluded that group norm as regards humanized care is present in all the NICU staff members (Costa & Padilha, 2011).

Peer reinforcement develops out of social interactions between the NICU staff. This is the second method used to sustain a “culture of silence” in our study. A study on qualitative examination of changing practice in the NICU has reported that three themes that influence change in practice are human resources, organizational structure, and communication (Stevens, Lee, Law, & Yamada, 2007). A multidisciplinary team of neonatologists, otolaryngologists, audiologists, social scientists, and nursing staff being involved in the evolution of the NsRP was a facilitating factor in our study. Similar findings have been noted in another study which has examined the factors influencing decision making regarding advocacy (Monterosso et al., 2005). Social interactions during group discussions have been used to explore the influences of context in altering behavioral practices about pain in the NICU (Stevens et al., 2011). The themes that emerged from their study were culture of collaboration and support for evidence-based practice, threats to autonomous decision making, and complexities in care delivery. All this reinforce the findings of our study about social interactions dynamically shaping the “culture of silence” created in the NICU.

A mixed methods approach study consisting of measuring noise using a sound level meter (Quantitative measure) and in-depth interview (Qualitative method) to examine the perceptions of nurses about the source of noise in the NICU has reported that the nursing staff are aware of high noise levels in the NICU (Darcy, Hancock, & Ware, 2008). The quantitative part of this study has been done by three studies in the Indian context (Livera et al., 2008; Ramesh et al., 2009; Vivek, Soodan, Girish, Vishal, & Nair, 2005). In our study, it was also seen that awareness momentum and percolation causing an evolution of adherence gets created by the initial establishment phase of the NsRP. These form the evidence base for the creation of the “culture of silence.”

Our study has concluded that the main strategy employed by the NICU staff to ensure adherence to behavioral modification components of the NsRP was by “sustaining a culture of silence in NICU during nonemergency situations.” The “culture of silence” ongoingly reconditions the behavior of the existing staff and conditions new staff to adhere to behavioral modifications. This model can be used as a template for other NICUs in resource-limited settings to employ similar strategies to establish context specific and effective NsRPs.
